# Colorectal Dysplasia and Cancer Surveillance in Ulcerative Colitis

**DOI:** 10.3390/diseases9040086

**Published:** 2021-11-19

**Authors:** Emily Reznicek, Mohammad Arfeen, Bo Shen, Yezaz A. Ghouri

**Affiliations:** 1 Department of Medicine, Division of Gastroenterology and Hepatology, University of Missouri School of Medicine, Columbia, MO 65212, USA; 2 Department of Gastroenterology, Franciscan Health, Olympia Fields, IL 60461, USA; 3 Interventional IBD Center, Department of Medicine and Surgery, Columbia University Irving Medical Center/New York Presbyterian Hospital, New York, NY 10032, USA

**Keywords:** inflammatory bowel disease, ulcerative colitis, cancer, colitis-associated neoplasia, surveillance, chromoendoscopy

## Abstract

Ulcerative colitis (UC) is a risk factor for the development of inflammation-associated dysplasia or colitis-associated neoplasia (CAN). This transformation results from chronic inflammation, which induces changes in epithelial proliferation, survival, and migration via the induction of chemokines and cytokines. There are notable differences in genetic mutation profiles between CAN in UC patients and sporadic colorectal cancer in the general population. Colonoscopy is the cornerstone for surveillance and management of dysplasia in these patients. There are several modalities to augment the quality of endoscopy for the better detection of dysplastic or neoplastic lesions, including the use of high-definition white-light exam and image-enhanced colonoscopy, which are described in this review. Clinical practice guidelines regarding surveillance strategies in UC have been put forth by various GI societies, and overall, there is agreement between them except for some differences, which we highlight in this article. These guidelines recommend that endoscopically detected dysplasia, if feasible, should be resected endoscopically. Advanced newer techniques, such as endoscopic mucosal resection and endoscopic submucosal dissection, have been utilized in the treatment of CAN. Surgery has traditionally been the mainstay of treating such advanced lesions, and in cases where endoscopic resection is not feasible, a proctocolectomy, followed by ileal pouch-anal anastomosis, is generally recommended. In this review we summarize the approach to surveillance for cancer and dysplasia in UC. We also highlight management strategies if dysplasia is detected.

## 1. Introduction

Colorectal cancer (CRC) is the third most common cancer and the second most common cause of cancer-related death in the United States [1]. In addition to human lives lost, CRC boasts a prominent financial burden of 11% of all cancer treatment costs and an annual price tag of 14.1 billion USD in national spending [2]. Approximately half of CRC cases are postulated to be preventable and/or manageable through risk modification and screening [3]. There are multiple modifiable risk factors related to the development of CRC. However, the presence of ulcerative colitis (UC) and the subsequent progression to colitis-associated neoplasia (CAN) pose unique challenges regarding dysplasia surveillance and management.

According to the Centers for Disease Control (CDC), the lifetime risk of CRC in the general US population is approximately 4% [3]. In contrast, the risk of CAN in patients with UC is estimated to be between 8% and 18% after 30 years of disease and may be 4 times higher for patients with comorbid primary sclerosing cholangitis (PSC) [4,5,6,7]. The association between UC and CAN is thought to be due to long-standing inflammation based on the observation that cancer-risk parallels disease severity and duration [8,9].

Most CRCs in UC are suspected to arise from inflammation-associated dysplasia rather than sporadic adenomas; therefore, alternative surveillance recommendations have been developed for this patient population. All current guidelines emphasize the role of endoscopy for the early detection of dysplasia and cancers [9,10,11,12]. While colonoscopy is considered the gold-standard test for CRC screening in the general population, it is the only modality recommended for CAN screening and may be performed using targeted biopsies with chromoendoscopy (CE) or random biopsies with white-light endoscopy (WLE). Compliance with initial CAN screening is relatively high among UC patients at roughly 90%; however, there is a decline on subsequent examinations, with 76% to 78% of patients being up to date on colonoscopy. This decline is still better when compared to the 68.8% of the general population who are up to date on their screening colonoscopy for colon cancer [13,14,15]. Several considerations limit guideline adherence, including patient and physician factors. Patients frequently cite transportation, finances, and discomfort with the procedure or bowel preparation as barriers to colonoscopy [16]. In particular, UC patients may be reluctant to undergo additional colonoscopies given the frequency required, especially while in remission. General practitioners report uncertainty in caring for UC patients despite being expected to manage the disease [17,18]. Additionally, gastroenterologists inconsistently follow surveillance guidelines, largely due to debates surrounding efficacy and cost-effectiveness [14,19].

The purpose of this article is to review recent studies and the latest guidelines regarding dysplasia surveillance in UC which, if left unaddressed, can lead to the subsequent development of CAN. We describe the pathogenesis of CAN, surveillance modalities and intervals, and the management of dysplasia and neoplasia.

## 2. Pathogenesis of Colitis-Associated Neoplasia

Colitis-associated neoplasia is thought to be the result of chronic inflammation, which induces changes in epithelial proliferation, survival, and migration via the effect of various chemokines and cytokines [20]. While sporadic CRC develops from one or two foci of dysplasia or adenoma, CAN is believed to develop from multiple dysplastic foci, by which chronically inflamed mucosa produces a field change of molecular alterations and, eventually, histologic changes [20]. Abnormal or disordered growth of cells is termed dysplasia and signifies a precancerous change of the mucosa. Dysplasia is subdivided into low-grade dysplasia (LGD) and high-grade dysplasia (HGD). LGD is characterized by cytological changes, such as nuclear atypia, and carries a relatively low risk for malignant transformation. In contrast, HGD demonstrates cytologic and architectural changes, such as loss of polarity or cribriform gland formation, and is a higher risk for malignant transformation. Occasionally, biopsies will return as indefinite for dysplasia if acute inflammation is present, which can mask or mimic dysplastic changes.

A whole-exome analysis of sporadic CRC and CAN from The Cancer Genome Atlas demonstrated a distinct mutation profile in patients with CAN. Although both exhibit a similar frequency of acquired genetic abnormalities, such as chromosomal instability (CSI) and microsatellite instability (MSI), there are notable differences in the timing and frequency of sequential anomalies. In general, p53 mutations tend to appear often and early in CAN while APC mutations arise later. K-RAS mutations appear infrequently. Alternatively, APC and K-RAS mutations occur often and early in sporadic CRC, whereas p53 mutations develop later in the course of carcinogenesis [21].

The longstanding infiltration of inflammatory cells results in the production of pro-inflammatory cytokines (IL-1, IL-6, TNF-α) and chemokines, which activate nuclear transcription factors (NF-kB and STAT3) to maintain inflammation and promote carcinogenesis via the loss of the p53 tumor suppressor gene and the activation of NF-kB and STAT3. Loss of p53 promotes unchecked cell growth and inhibits apoptosis while augmenting cytokine-mediated DNA damage. Active transcription factors stimulate the production of cytokines and reactive oxygen species (ROS) and enhance MYC proto-oncogenic expression. This feedback loop of inflammation, DNA damage, and cell growth ultimately leads to the remodeling of the extracellular matrix and metastasis [20,22,23]. Lastly, the overgrowth of genotoxic microorganisms has been shown to implicate dysbiosis as a risk factor for carcinogenesis via the decreased production of anti-inflammatory cytokines such as IL-10. Given the high prevalence of gut dysbiosis in UC, further studies are warranted to study these preliminary findings [24].

The pathogenesis of CAN involves a complex system of interactions between the environment, gut microbiome, and genetics. Dysregulation of these systems leads to a chronic state of inflammation, which induces cellular and architectural changes over time and ultimately develops into CAN.

## 3. Surveillance Modalities

Given the high risk of CAN arising from dysplasia in patients with UC, endoscopic screening is recommended for all patients with left-sided (approximately one-third of colon involved) or pan-colonic disease, starting 8 years after symptom onset. Very few cancers develop prior to 8–10 years after symptom onset; therefore, it is not cost-effective to screen patients sooner. The universal exception is PSC, for which patients should undergo screening colonoscopy at the time of diagnosis and every year thereafter [9,10,11,12]. Patients with ulcerative proctitis and proctosigmoiditis do not have a significantly increased risk of CAN compared to the general population and should follow routine guidelines for CRC screening [25,26].

Unlike sporadic CRC, there is no validated imaging or lab tests for CAN screening. Colonoscopy is the cornerstone of prevention and management of dysplasia in patients with UC. In general, there are three modalities for CAN screening: standard-definition white-light endoscopy (SD-WLE), high-definition white-light endoscopy (HD-WLE), and CE (which here refers to dye-based CE). As endoscopic technology continues to evolve, societal guidelines are updated to represent the latest tools and data to help guide surveillance strategies for patients with UC. Specifically, the SCENIC (Surveillance for Colorectal Endoscopic Neoplasia Detection and Management in Inflammatory Bowel Disease Patients International Consensus) guidelines were established in 2015 to address surveillance and management strategies of dysplasia in inflammatory bowel disease (IBD) (Table 1) [9,10,27,28].

### 3.1. Conventional White-Light High-Definition Colonoscopy

It was previously thought that dysplasia and CAN arise from molecular alterations in normal-appearing mucosa, which expand and form premalignant patches and then dysplasia and cancer, also known as “field effect”, and that random biopsies would help identify at-risk mucosa [29]. More recently, it has been noted that 58% to 94% of dysplastic lesions may be identified with SD-WLE; however, this yield increases with the addition of chromoendoscopy or use of HD-WLE [30,31]. Since SD-WLE regularly yields inferior detection rates compared to HD-WLE and CE, targeted and random biopsies every 10 cm for a total 33 or more biopsies are recommended. When available, HD-WLE or CE are preferred, and targeted biopsies are sufficient to detect dysplasia [9,10,11,12]. Additional random biopsies are of low yield and may not be worth the extra time and cost [32].

High-definition (1080 pixels or higher) endoscopy is unanimously preferable to standard-definition (480 pixels) endoscopy. If HD-WLE is not available, CE should be incorporated into the SD-WLE. However, there is debate amongst gastroenterologists as to whether the use of HD-WLE should include CE. When CE is performed, each colonic segment is evaluated by HD-WLE, followed by segmental withdrawal and the reintroduction of the colonoscope, as well as spraying with dye, which is usually diluted methylene blue or indigo carmine solution. The methylene blue dye is used at 0.04% concentration; this is created by mixing one vial (10 mL) of 1% methylene blue with 240 mL of water [33]. Generally, two vials are used, the dye is therefore mixed in about 480 mL of water. After the spraying of the methylene blue solution, one minute is usually given for the uptake of dye before examination. Similarly, an indigo carmine solution of 0.03% concentration is used for CE [33]. This is generated by mixing 0.8% indigo carmine 10 mL solution in 250 mL of water; usually, two vials are mixed in 0.5 L of water. The solution accumulates in the colonic pits and ridges, and the sprayed mucosa can be examined almost immediately.

Although high-definition CE (HD-CE) is preferred to HD-WLE, as per the SCENIC guidelines, the quality of evidence is low as studies inconsistently favor HD-CE [34,35]. More recently, a 2019 meta-analysis of randomized control trials demonstrated that HD-WLE was comparable to chromoendoscopy for identification of dysplasia [36]. There are also considerations of additional time, preparation, and training associated with the use of CE, as well as a lack of procedure code in the United States. It is unclear how this information will affect future guidelines.

### 3.2. Image-Enhanced Colonoscopy

Alternate methods to colonoscopy and dye-based CE have been studied for the detection of dysplasia; however, these are not yet supported by major GI society guidelines. Narrow-band imaging (NBI) [Olympus, Toyko, Japan] utilizes optical digital techniques to filter white light towards blue and green to enhance the visualization of vascular patterns and architecture of colonic mucosa. This is a form of virtual CE. Although studies have not demonstrated a meaningful change in dysplasia detection rates with NBI, it may be useful in conjunction with magnification endoscopy, as a better means of characterizing dysplastic lesions [37]. Newer forms of virtual chromoendoscopy techniques, such as Fuji intelligent color enhancement (FICE) [Fujinon, Fujifilm, Toyko, Japan], i-scan (Pentax, Tokyo, Japan) and confocal laser endomicroscopy (CLE), are emerging as options for the detection of dysplasia in UC. However, relevant data are limited about their utility in clinical practice, and studies are ongoing [38,39].

### 3.3. Fecal Markers

Fecal tumor markers have been suggested as alternatives to conventional endoscopy and include detecting fecal DNA methylators (EYA4, BMP3, NDRG4, SLIT2), micro-RNA, calprotectin, and M2PK. Although stool studies are an attractive option due to their convenience and safety, these are more suitable for predicting advanced lesions and may not be sensitive for early stages of dysplasia [38]. A comparison of various endoscopic modalities used for surveillance has been summarized in Table 2 [36,40].

## 4. Surveillance Strategies

As previously stated, chronic intestinal inflammation can lead to dysplasia, usually LGD or HGD, both of which carry a major risk for developing CAN. De Jong et al. in their nationwide cohort study using the Dutch National Pathology Registry concluded that in their large cohort of IBD patients with LGD the cumulative incidence of advanced neoplasia was 21.7% after 15 years. Other independent risk factors for developing advanced neoplasia were age > 55 years, male gender, and follow-up at a tertiary IBD referral center [41]. Additionally, there was a component of diagnostic error in the biopsies revealing LGD, with data showing a significant degree of intra- and inter-observer variability in determining indefinite for dysplasia and LGD [42].

The 2019 American College of Gastroenterology (ACG) guidelines recommend initiating surveillance for CAN 8 years after index diagnosis. If the initial screening exam is negative for dysplasia, then repeat examinations every 1–3 years are recommended based on the combined risk of CAN and previous endoscopic and histologic findings. In the case of UC-associated neoplasia, they recommend that the slides be reviewed by an experienced GI pathologist and neoplastic findings be reviewed by a second experienced GI pathologist; this is also suggested by the European Crohn’s and Colitis Organization (ECCO) [9,27]. The more recent American Gastroenterology Association (AGA) best practice advice statement, released in 2021, suggests that this period of follow-up surveillance can be 1 to 5 years [28]. This is dependent on the risk of developing CRC, the burden of inflammation, family history of CRC, history of PSC, the detection of prior dysplasia, and the quality and frequency of previous surveillance colonoscopies. In De Jong et al.’s analysis of the Dutch Registry they showed that approximately 25% of all advanced neoplastic lesions were detected within 1 year of the detection of LGD. Additionally, about 25% of patients who had a colectomy within 1 year of LGD detection had metachronous advanced neoplasia in their colectomy specimens [41]. Thomas et al. in their systematic review also found that 22% of the patients with LGD who underwent colectomy had synchronous CRC [43]. They suggested that the first surveillance colonoscopy should be performed within 1 year of LGD detection [41]. The current guidelines differ on the recommended surveillance intervals for colonoscopy, with the current United States guidelines recommending 1–3 or 1–5 years [9,28]. The ECCO consensus statement recommends repeat surveillance based on the risk of developing dysplasia. The statement proposes three risk-categories—high, intermediate, and low risk—and suggests a colonoscopy interval of 5 years, 2 to 3 years, and 1 year, respectively, for each category [27]. High risk includes individuals with PSC, dysplasia or stricture detected within the past 5 years and severe active colitis, intermediate risk includes individuals with mild to moderate colitis, and low risk includes individuals without any of these features.

However, in the case of patients with a history of dysplasia, the consensus is that they should undergo annual surveillance with colonoscopy [12]. In the case of invisible LGD (i.e., dysplasia found on random biopsies, as opposed to a target biopsy of a mucosal lesion or a polyp) detected on HD-WLE, the current American Society of Gastrointestinal Endoscopy (ASGE) guidelines recommend a repeat evaluation with chromoendoscopy by experienced endoscopists, during which random and targeted biopsies should be obtained; surface CE may also be utilized in highlighting mucosal aberrancies. This may also assist in determining the endoscopic resectability of dysplastic lesions [12].

The management of indefinite dysplasia (IND) remains controversial. A lesion is labeled to have IND when, upon expert review of its histology, it does not exhibit the necessary changes to be diagnosed as LGD but has some features which are concerning for dysplasia. This is usually seen in the setting of active colitis, when the atypia resulting from the active inflammatory process can interfere with the definitive diagnosis of dysplasia. Pekow et al. in their small single-center chart analysis of patients with IND and LGD showed a low rate of conversion to HGD or CRC [44]. Conversely, Lai et al. showed a conversion rate from IND to HGD or CRC of 1.5 cases per 100 person-years, suggesting that IBD patients with IND remain at significant risk for HGD or CRC and should be closely surveyed [45]. Van Schaik et al. in their review of the Dutch National Pathology Registry were able to demonstrate a 19% conversion rate from IND to advanced neoplasia after a median time of 24 months [46]. In a 30-year analysis of colonoscopic surveillance for neoplasia in UC, Rutter et al. showed that 4% of their patients with IND developed carcinoma, and 22% developed LGD after 9 years of follow-up [6]. Data from another large group showed that the 5-year conversion rate from IND to HGD or CRC was 9% [47]. Based on the data and the guideline recommendations it would be reasonable to recommend a repeat colonoscopy within 6–12 months with either HD-WLE or surface CE and annual surveillance subsequently. Additionally, it may be worth considering the escalation of treatment to ensure histologic remission so that future biopsies may present a clearer picture without the atypia associated with active disease.

As per the current ASGE guideline, SCENIC international consensus statement, ECCO consensus statement, ACG guideline, and AGA clinical practice update on dysplasia in UC, dysplasia detected on biopsy with a discrete lesion should be resected endoscopically if possible. If complete endoscopic resection is achieved, the patient should be followed with surveillance colonoscopies at shortened intervals (i.e., 1 month, 6 months, and 1 year) with biopsies obtained from the base of the resection site. In these patients, total proctocolectomy may not be necessary. However, patients with invisible dysplasia (e.g., dysplasia detected on random biopsies or not from a discrete lesion) should be referred for repeat HD-WLE endoscopy with surface CE by an endoscopist experienced in IBD surveillance [10].

Regarding patients with a J-pouch and an ileal pouch-anal anastomosis (IPAA) there is limited guidance regarding frequency and timing of surveillance endoscopy or if it is necessary at all once the pouch is created. The ECCO guidelines recommend early pouchoscopy in symptomatic patients with pouch dysfunction to distinguish between pouchitis and other conditions such as CMV colitis, ischemic pouch, or *Clostridioides difficile* infection [27]. Additionally, an annual pouchoscopy is recommended in patients with risk factors such as a history of neoplasia and primary sclerosing cholangitis [27]. They do not recommend a specific follow-up strategy in asymptomatic patients. Samaan et al. in their multi-national retrospective cohort study identified two cases of adenocarcinoma in the rectal cuff of low-risk patients [48]. They concluded that it may be beneficial to perform pouchoscopy 1 year post-operation, with biopsies to further risk stratify patients based on clinical factors alone. More recently, an analysis of the Cleveland Clinic ileoanal pouch anastomosis database showed 0.14% of procedures showed biopsy-proven neoplasia, 0.07% with LGD, none with HGD, and 0.06% with invasive adenocarcinoma. Of the patients with adenocarcinoma all were symptomatic at the time of pouchoscopy, had a negative surveillance pouchoscopy within two years prior to their diagnosis of adenocarcinoma, and had palpable masses on digital rectal examination [49]. They concluded that surveillance pouchoscopy in asymptomatic patients is not recommended based on the rare occurrence of neoplasia.

## 5. Management of Dysplasia and Cancer

**Figure 1:**
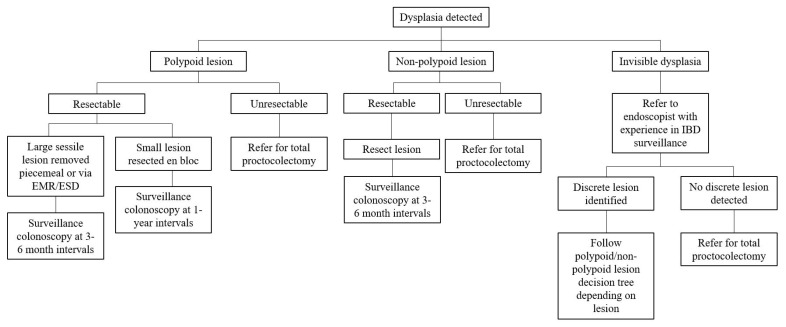
Approach to management of colorectal dysplasia and cancer in patients with ulcerative colitis.

Historically, surgery has been the mainstay of treatment for dysplasia and CAN in UC patients. In recent years advanced endoscopic resection techniques have become a first-line approach in their management, thereby sparing these patients from undergoing total proctocolectomies. Depending on the resectability of the lesion, it is a viable option to endoscopically resect dysplastic lesions and then follow up with enhanced endoscopic surveillance at shorter intervals. We further describe this strategy in addition to the traditional surgical alternatives for the management of dysplasia and cancer in patients with UC (Figure 1).

### 5.1. Endoscopic Management

Endoscopically visible lesions within an area of colitis should be evaluated for resectablity, with the knowledge that resecting lesions in areas of active inflammation can be more challenging. As per the ASGE guideline and AGA clinical practice update, endoscopically visible lesions that are clearly demarcated without evidence of submucosal invasion should be considered for endoscopic resection [12,28]. When the expertise is available, these lesions should be resected en bloc to confirm complete histologic resection; this may necessitate a referral to centers with advanced polypectomy experience. Additionally, the ASGE guidelines recommend that, after mucosal resection is completed, biopsies of the surrounding mucosa should be obtained to ensure that the margins of the lesion are clear [12]. In lesions with signs of submucosal invasion such as depressions or failure to lift after submucosal injection, further evaluation with techniques using endoscopic ultrasound or confocal endomicroscopy can be performed to determine resectability.

A meta-analysis of UC patients who underwent follow-up colonoscopy after endoscopically resected polypopid dysplasia revealed that the pooled incidence of cancer was 5.3 cases/1000 years of patient follow up, and the pooled incidence of dysplasia was 65 cases/1000 patient-years [50]. Based on this data, it is reasonable to conclude that resection and surveillance is a reasonable strategy; but the rate of dysplasia is approximately 10 times higher than CRC and, therefore, these patients should be closely followed with surveillance colonoscopies. Hurlstone et al. investigated the role of endoscopic mucosal resection (EMR) for flat neoplastic lesions in the setting of UC. They compared rates of CRC development, resection efficacy, metachronous lesion rates, and post-resection recurrence rates in patients with colitis-associated Paris class 0-II and class I adenoma-like mass lesions undergoing EMR against sporadic controls. They did not find any statistical differences between their study groups; however, they noted there was a significantly higher prevalence of Paris class 0-II lesions in the UC group and a higher recurrence rate of laterally spreading tumors in the colitis group than in the control groups [51]. This suggests that in patients with flat lesions, EMR remains a viable option. The Australian Colonic Endoscopic resection study group performed a prospective, multicenter, observational study to assess outcomes of patients with UC who underwent EMR for sessile polyps greater than 20 mm in size. The most observed lesion was the Paris class 0-IIa granular type with a 1.4% rate of submucosal invasion, and EMR was effective at achieving complete resection of the polyp in a single session in 89.2% of patients. Risk factors for lack of efficacy of EMR included prior attempts at EMR and ileocecal valve involvement. Predictors of recurrence after EMR included a lesion size greater than 40 mm and the use of argon plasma coagulation (APC). In their dataset, no patients died after undergoing EMR, and 83.7% of patients avoided surgery [52].

A study by Smith et al. on endoscopic submucosal dissection (ESD) in patients with mass-like adenomas in the setting of UC, and usually with mucosal fibrosis, demonstrated that it may be a feasible option. Of the 69 patients in the study, en bloc resection was successfully performed in 78% of cases, whereas 7% required a piecemeal resection. R0 resection was achieved in 94% of lesions that were removed en bloc. Complication rates were modest with a 3% perforation rate and a 10% bleeding rate. After a median follow up period of 18 months, the cure rate for patients who underwent ESD-assisted EMR was 98% [53].

### 5.2. Surgical Management

For patients who develop CAN, the American Society of Colon and Rectal Surgeons recommends offering total proctocolectomy with or without IPAA as a valid approach for management, provided there is no evidence of metastasis [54]. Others have suggested an approach where the patient undergoes an abdominal colectomy with ileostomy followed by a restorative proctectomy after an observation period of 12 months of monitoring for disease recurrence [55].

In an analysis of the Danish Cancer Registry, Mark-Christensen et al. matched 1723 patients who had an IPAA with 8615 individuals from the general population, in a median follow-up of 12.9 years. They found two cases of cancer in the pouch (0.12%) as opposed to 38 cases of intestinal cancers (0.45%) in the general population group. However, they did note an increased risk of hepatobiliary cancer for patients with IPAA; with an incidence rate ratio of 13; interestingly, half the population with IPAAs that developed hepatobiliary cancers had underlying PSC [56]. Guidance regarding the management of pouch neoplasia is sparse. There is some evidence that endoscopic resection of LGD of the pouch with surveillance is a viable option [57]. A pouch with HGD or carcinoma typically requires surgical resection with surgical mucosectomy. Pouch advancement, pouch excision, and complete proctectomy have all been proposed as potential options [58].

### 5.3. Future Directions

The current standard for the surveillance for dysplasia and neoplasia in UC is colonoscopy. With the discovery of molecular biomarkers, there is a potential for risk stratifying patients and ultimately instituting less intense surveillance regimens for low-risk patients. Module-1 of the ENDCAP-C study showed that their panel of five markers (SFRP2, SFRP4, WIF1, APC1A, and APC2) was accurate in detecting pre-cancerous and invasive neoplasia and dysplasia. Their four-marker panel (APC1A, SFRP4, SFRP5, and SOX7) showed less accuracy at predicting bowel neoplasia [59]. Stool DNA tests such as Cologuard have helped to increase CRC screening in certain average risk populations but have not yet been validated in UC patients. Similarly, colon capsule endoscopy has been used for CRC screening in average-risk individuals but remains an invalid option for dysplasia surveillance due to its inability to obtain tissue samples [60]. The advancements in molecular genetics and non-invasive screening technologies will hopefully continue to evolve and improve the quality for dysplasia screening and surveillance in the UC population.

## 6. Conclusions

Ulcerative colitis-associated dysplasia and cancer are complications that can be detected early in their development and treated with endoscopic techniques such as EMR and ESD. Surgery, which has been a mainstay for managing these conditions, is a far more aggressive option and can be avoided when these lesions are endoscopically resectable. Screening with HD-WLE, especially with concomitant use of CE, can improve the quality of detecting these lesions. Multiple GI societies have proposed guidelines and consensus statements to help direct practicing gastroenterologists to appropriately survey the at-risk UC patients for dysplasia and CAN. Awareness about various methods of surveillance and management of dysplasia and CAN is necessary to prevent any delay in diagnosis of these conditions which may necessitate far more aggressive treatment in the form of major bowel surgery, and in some cases, may mean such conditions are not be amenable to any curative therapy.

## Figures and Tables

**Table 1:** A concise summary of selected multiple GI society guidelines on the endoscopic surveillance and management of colorectal dysplasia in ulcerative colitis (these are interpretations of the guidelines published by multiple GI societies; please refer to the original published guidelines for further details [9,10,27,28]).

SCENIC (2015)	AGA (2021)
HD-WLE recommended above SD-WLE. Chromoendoscopy is recommended rather than WLE. NBI exam is not suggested in place of WLE or chromoendoscopy. After removal of endoscopically resectable lesions, surveillance colonoscopy is preferred over colectomy. If invisible dysplasia is detected on random colonic biopsies, then refer to an endoscopist with expertise in using chromoendoscopy with HD-WLE.	Lesions should be described as polypoid (2.5 mm tall), non-polypoid (< 2.5 mm), or invisible (detected on non-targeted biopsy) using a modified Paris Classification. Lesions should be described based on size, morphology, clarity of borders, presence of ulceration, location, presence within an area of past or current colitis, perceived completeness of resection, and whether any special techniques were used to visualize the lesions. Targeted biopsies should be obtained where mucosa appears suspicious for dysplasia or is inexplicably different from surrounding mucosa. Endoscopic resection is preferred to biopsies when lesions are clearly demarcated without stigmata of invasive cancer or submucosal fibrosis. Biopsies of the margins and surrounding mucosa are not required unless there are concerns about the completeness of resection. Dye spray chromoendoscopy should be considered especially if an SD endoscope is used or if there is history of dysplasia. Virtual chromoendoscopy is a suitable alternative to dye spray chromoendoscopy while using HD endoscope. Quadrantic biopsies every 10 cm should be taken from flat colorectal mucosa in areas previously affected by colitis when WLE is used without chromoendoscopy. Non-targeted biopsies are not routinely required if chromoendoscopy is used with a HD endoscope but should be considered for patients with history of dysplasia or PSC. All clearly delineated dysplastic appearing lesions without stigmata of invasive cancer or significant submucosal fibrosis should be considered for endoscopic resection. Findings of invisible dysplasia should prompt a repeat examination by an experienced endoscopist using HD dye spray chromoendoscopy under optimized viewing conditions with extensive non-targeted biopsies in area of prior dysplasia if no lesion is seen. If unresectable visible dysplasia or invisible multifocal or high-grade dysplasia is detected, then refer for colectomy. Patients with resectable lesions or if histologic dysplasia is not confirmed on high-quality dye spray chromoendoscopy, continued endoscopic surveillance at shortened intervals is appropriate. Targeted biopsies of concerning pseudopolyps are appropriate; removal and sampling of all lesions is not required. Surgery should be a last resort to manage colorectal cancer risk in the setting of severe pseudopolyposis. Dye spray chromoendoscopy should not be used to detect flat or subtle lesions within a field of pseudopolyps.
**ECCO (2017)**	
Colonoscopic surveillance is best preformed when UC is in remission. Chromoendoscopy with targeted biopsies, or quadrantic biopsies every 10 cm and targeted biopsies of any visible lesion should be performed if WLE is used. High-definition endoscope should, preferably, be used. Low-grade or high-grade dysplasia, if detected, should be confirmed by a second pathologist who is an expert in GI pathology. Continued surveillance is reasonable for endoscopically resectable lesions, if complete resection can be achieved with no evidence of non-polypoid or invisble dysplasia present elsewhere in the colon. If non-polypoid dysplasia is not fully excised, then patient should be referred for colectomy. Polyps that occur proximal to segments with active UC should be managed like sporadic adenomas.	
**ACG (2019)**	
During colonoscopy, identified lesions that are raised or have abnormal pit patterns should be targeted for biopsy and placed in separate jars from other segmental biopsies. Most neoplasia in UC is visible with SD/HD-WLE. It is unclear if segmental random biopsies of colon are required during a surveillance exam. Neoplastic findings should be reviewed by a second experienced GI pathologist. When dysplasia of any grade has been completely removed, proctocolectomy may not be necessary, and initial surveillance at shortened intervals must be performed. When dysplasia is not resectable or is multifocal, then refer to patient for proctocolectomy. Patients with extensive inflammatory polyps may not be able to have adequate surveillance and may require more frequent surveillance or may undergo surgery. Patients with UC-associated dysplasia who undergo subsequent surveillance may benefit from dye spray chromoendoscopy during the first follow-up exam. Fecal DNA and CT colonography are not recommended for screening or surveillance of UC-associated neoplasia.	

HD-WLE: high-definition white-light endoscopy, SD-WLE: standard definition white-light endoscopy, UC: ulcerative colitis, PSC: primary sclerosing cholangitis.

**Table 2:** A comparison of various surveillance modalities used for detecting colorectal dysplasia in patients with ulcerative colitis [36,40].

	Advantages	Disadvantages
SD-WLE with random biopsies	Widely available	Cost effective Longer procedure time Lower quality images
HD-WLE with targeted biopsies	High quality images Widely available High dysplasia detection rate	Higher cost
Dye-based CE with targeted biopsies	Cost effective due to fewer biopsies and longer surveillance intervals Highest dysplasia detection rate	Higher cost Longer procedure time Specialized equipment required Additional training required No procedure code in the United States
WLE with NBI	Widely available	No added benefit to WLE Longer procedure time
Fecal tumor markers	Non-invasive Low risk to patient	Limited data Detects advanced lesions only Not widely available

HD-WLE: high-definition white-light endoscopy, SD-WLE: standard definition white-light endoscopy, CE: chromoendoscopy, NBI: narrow band imaging.
